# Properties and Applications of Quaternary Ammonium Gemini Surfactant 12-6-12: An Overview

**DOI:** 10.3390/molecules28176336

**Published:** 2023-08-30

**Authors:** Bogumił Brycki, Adrianna Szulc, Justyna Brycka, Iwona Kowalczyk

**Affiliations:** 1 Department of Bioactive Products, Faculty of Chemistry, Adam Mickiewicz University Poznan, Uniwersytetu Poznańskiego 8, 61-614 Poznan, Poland; adaszulc@amu.edu.pl (A.S.); iwkow@amu.edu.pl (I.K.); 2 MDA Sp. z o.o., Wolczynska 18, 60-003 Poznan, Poland; biuro@mda.org.pl

**Keywords:** gemini surfactants, 12-6-12, synthesis, properties, antimicrobial activity, applications

## Abstract

Surfactants are amphiphilic molecules and one of the most versatile products of the chemical industry. They can be absorbed at the air–water interface and can align themselves so that the hydrophobic part is in the air while the hydrophilic part is in water. This alignment lowers the surface or interfacial tension. Gemini surfactants are a modern variety of surfactants with unique properties and a very wide range of potential applications. Hexamethylene-1,6-bis(*N*-dodecyl-*N*,*N*-dimethylammonium bromide) is one such representative compound that is a better alternative to a single analogue. It shows excellent surface, antimicrobial, and anticorrosion properties. With a highly efficient synthetic method and a good ecological profile, it is a potential candidate for numerous applications, including biomedical applications.

## 1. Introduction

Interactions at the interface are of fundamental importance in chemistry, physics, and biology. The thermodynamic parameters of these interactions can be modulated using surfactants. Owing to their amphiphilic structure, which comprises a hydrophilic and hydrophobic part, surfactants decrease the surface tension or interfacial tension between two liquids, a liquid and gas, or a liquid and solid. Hence, they can act as wetting, dispersing, and emulsifying agents. These properties enable the production of a large number of products required in household chemistry and cosmetic, pharmaceutical, agrochemical, petrochemical, textile, and paper industries.

The dynamic development of surfactant chemistry is a continuation of what was initiated by nature, which created the biosurfactants necessary for the functioning of living organisms. Biosurfactants usually refer to surfactants of microbial origin and comprise lecithin, rhamnolipids, sophorolipids, and emulsan [1,2]. Biosurfactants, in addition to their intrinsic role, have a wide range of technical applications. For instance, they can solubilise hydrocarbon contaminants and can be used in enhanced oil recovery [3,4]. Owing to their low toxicity and biodegradability, biosurfactants are extremely valuable products from the viewpoint of environmental protection. The global market size for biosurfactants reached a value of more than USD 2.33 billion in 2021 and is expected to grow at a compound annual growth rate (CAGR) of 5.8% between 2023 and 2028, reaching a projected value of USD 3.27 billion by 2027 [5,6]. Unfortunately, the number of available biosurfactants as well as the range of their applications does not meet the requirements expected from surfactants.

Currently, the widest groups of surfactants that meet the application requirements are nonionic, anionic, cationic, and amphoteric synthetic surfactants. The global demand for these surfactants, including soaps, exceeds 20 million tons per year [6]. The global surfactant market stood at a value of approximately USD 41.84 billion in 2022. The market is further expected to grow in the forecast period 2023–2030 at a CAGR of 4.6% to reach a value of USD 59.95 billion by 2030 [6]. However, the large volume of surfactants disposed into the environment, despite the wastewater treatment processes, is a serious burden and threat to the environment. Thus, owing to the increasing demand for surfactants, the development of new and more effective surfactants is extremely important.

**Figure 1:**
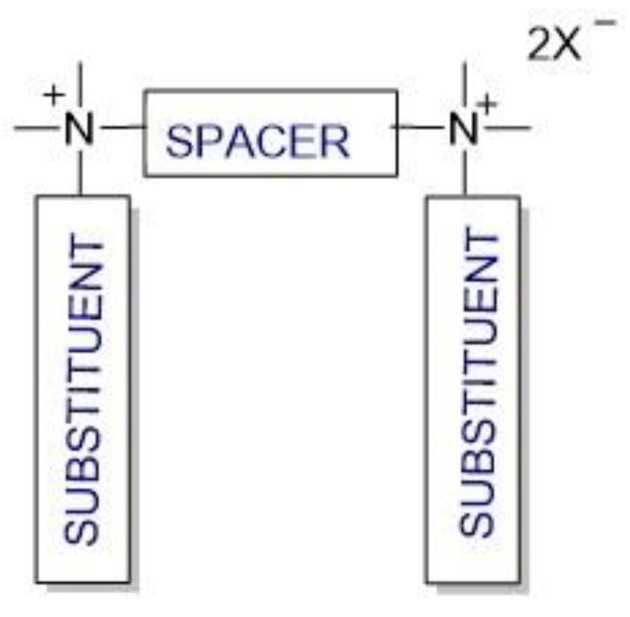
Structure of gemini surfactants.

Cationic gemini surfactants have emerged as a result of the research conducted in this field over the last several years. Gemini surfactants are compounds that are composed of two hydrophilic head groups and two hydrophobic tails linked by a spacer at the head groups or close to them (Figure 1). The spacers can be flexible (methylenes) or rigid (aromatic structures). The type of spacer (short or long, hydrophilic or hydrophobic) influences the shape of the micelles. The neutral charge of the molecule is retained in the presence of organic or inorganic counterions [7,8,9].

The critical micelle concentration (CMC), surface tension (γ), and minimal inhibitory concentration (MIC) of gemini surfactants are a dozen times lower than those of monomeric surfactants. The unique properties of gemini surfactants with a wide range of hydrophilic–lipophilic balance (HLB) render them a very useful and innovative material in chemistry (e.g., for corrosion inhibition, micellar catalysis, nanoparticle synthesis, preparation of supramolecular solvents, nanoemulsion preparation, and synthesis of precisely defined polymers) [10,11,12,13,14], medicine (as biocides, drug carriers, and capping agents for metal nanoparticles with biocidal properties or for preparing nonviral gene delivery systems and inducing protein conformational changes) [15,16,17,18,19,20], and optoelectronics (through a spatial network of well-dispersed molecules) [7]. Gemini surfactants are a modern solution for all areas that need surfactants, including households, detergents, personal care, institutional and industrial cleaning, food processing, plastics, paints and coatings, oilfield chemicals, petrochemistry, agricultural chemicals, adhesives, and textiles [20].

**Figure 2:**
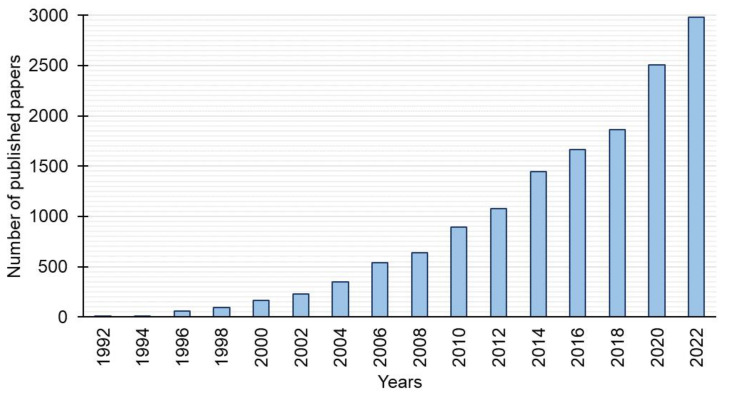
Number of papers published on gemini surfactants versus the year of publication (based on Scopus).

There is an increasing interest in gemini surfactants (Figure 2). Among the large number of gemini surfactants reported in recent years, hexamethylene-1,6-bis(*N*-dodecyl-*N*,*N*-dimethylammonium bromide) (12-6-12) deserves a special mention from the viewpoint of application. This review systematically presents the current studies devoted to the structure elucidation, synthesis, properties, and applications of 12-6-12, which can be a safer alternative to the surfactants used so far.

## 2. Structure and Synthesis

**Figure 3:**
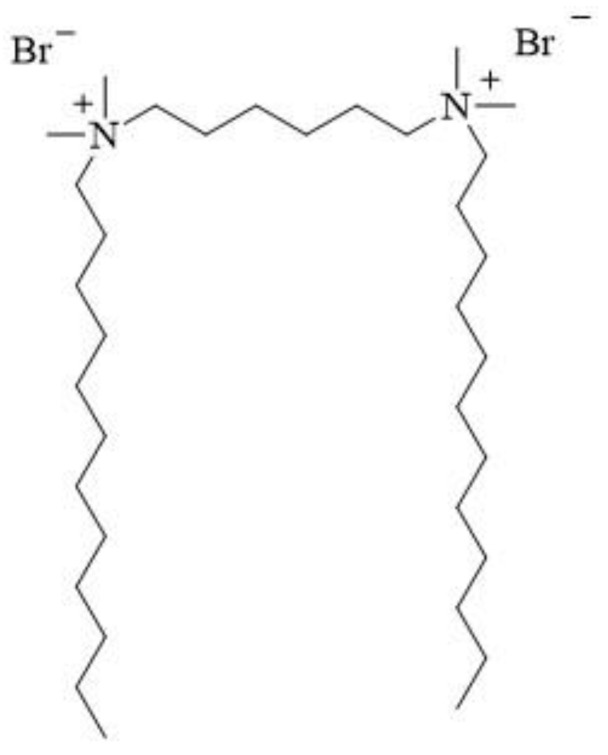
Chemical structure of 12-6-12.

12-6-12 consists of two *N*-dodecyl-*N*,*N*-dimethylammonium units connected with a chain of six methylene groups as a spacer. Bromine ions are present as counterions (Figure 3). This compound has been classified under chemical abstracts service (CAS) number 18507-15-8 and is a dimeric analogue of *N*-dodecyl-*N*,*N*,*N*-trimethylammonium bromide (DTAB), which is commonly used as a microbiocide.

**Figure 4:**
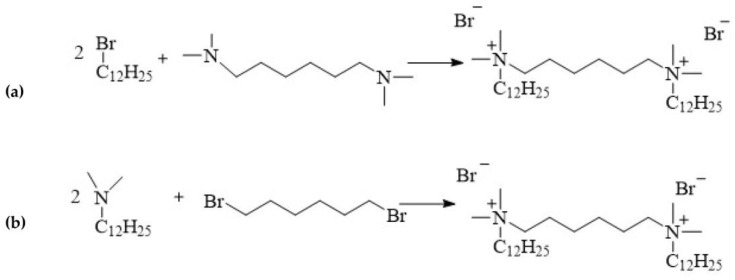
Synthesis of 12-6-12.

12-6-12 is one of the first compound to be classified as a double quaternary ammonium salt and defined as a gemini surfactant. This compound is synthesised via the quaternisation of amines, a process referred to as the Menschutkin reaction:

- alkylation of hexamethylene-bis(*N*,*N*-dimethylamine) with 1-dodecylbromide (Figure 4a);
- linking of *N*-dodecyl-*N*,*N*-dimethylamine with 1,6-dibromohexane (Figure 4b).

The synthesis of 12-6-12 was first reported in 1968 by Sindenko et al. [21]. Stoichiometric amounts of dodecylbromide and hexamethylene-bis(*N*,*N*-dimethylamine) were reacted in ethanol for the synthesis (yield 70%) [21]. Regardless of the synthetic pathway, the quaternisation reaction always follows the S_N_2 nucleophilic substitution mechanism. The rate of the reaction depends on the concentrations of both reagents, although there are reports of the use of excess amine [22,23,24,25] or bromide [26,27,28]. Typically, polar solvents such as alcohols (methanol [27,29], ethanol [30,31], isopropanol [32]), acetone [19,23,33], or acetonitrile [22,24,34] are used in the synthesis of 12-6-12. These reactions occur at the boiling point of the solvent. The type of solvent used determines the reaction time because S_N_2 reactions are the fastest in polar aprotic solvents such as acetonitrile. Replacing ethanol with acetonitrile reduces the reaction time from 24 to 5 h [25,35]. 12-6-12 can also be synthesised in solvent mixtures such as the acetonitrile/toluene mixture [36]. The reaction time can be shortened using microwave radiation [37]. However, the most economical and ecological approach is to synthesise 12-6-12 under stoichiometric conditions at room temperature without a solvent [38]. In this case, good yields of over 90% can be achieved using small amounts of reagents over a reaction time of 0.5 h [38]. Solvent-free synthesis can also be easily carried out on a large scale.

To obtain pure 12-6-12, the crude product can be crystallised from acetonitrile [35] or from dichloromethane–diethyl ether [39], acetone–methanol [40,41], ethanol–ethyl acetate [23], ethanol–diethyl ether [24], acetone–ethanol [21], and acetone–ethyl acetate [25] mixtures.

## 3. Analysis

**Figure 5:**
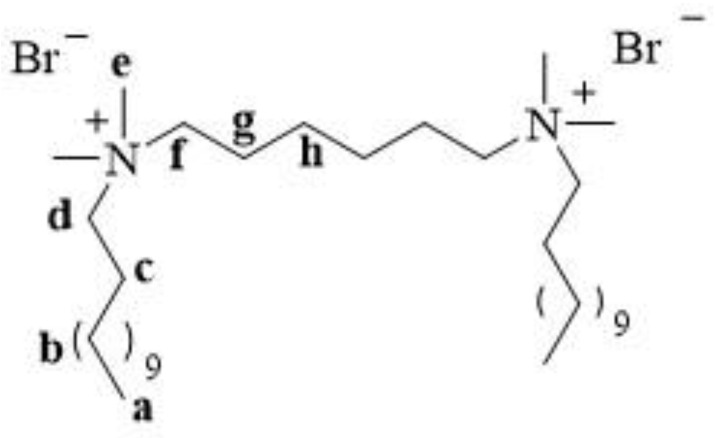
Structure and numbering of 12-6-12.

12-6-12 is a white water-soluble solid [26]. It melts with decomposition at 225–226 °C [28,30]. The structure of a compound is usually confirmed using proton and carbon nuclear magnetic resonance (^1^H and ^13^C NMR). The structure and numbering of 12-6-12 are shown in Figure 5.

In the ^1^H NMR spectra, signals from the protons of the terminal methyl groups of the long alkyl chains (a) were observed at the lowest ppm values. The protons of the methyl (e) and methylene groups (in spacer (f) and alkyl chains (d)) next to the quaternary nitrogen atom exhibited signals at the highest ppm values. Table 1 shows the ^1^H NMR chemical shifts for 12-6-12.

In the ^13^C NMR spectra, signals from the carbons of the terminal methyl groups of the long alkyl chains were observed at the lowest ppm values, similar to that in the ^1^H NMR spectra. Signals from the methylene groups in the alkyl chains, spacers, and methyl groups adjacent to the quaternary ammonium nitrogen appeared at the highest ppm values. Table 2 shows the ^13^C NMR chemical shifts of 12-6-12.

In the FTIR spectrum of 12-6-12, broad intense absorption bands corresponding to asymmetric stretching (ν_as_) and symmetric stretching (ν_s_) vibrations of the methyl and methylene groups were observed at 2980–2850 cm^−1^, while typical bands corresponding to deformation vibrations (δ) of the methyl and methylene groups appeared at 1490–1370 cm^−1^. At 720 cm^−1^, there was a typical band corresponding to the rocking vibrations (ρ) of the methylene groups derived from the long alkyl hydrocarbon chains. No stretching vibration bands for the N-H and O-H bonds were observed, which confirmed the purity of the compound [38].

Mass spectrometry is another analytical method for confirming the structure and purity of synthesised compounds. Currently, soft ionisation techniques, such as electrospray ionisation (ESI), are used for diagnostic purposes. Although methods leading to many decays and fragmentation ions for 12-6-12 have been published [43], they are currently of little diagnostic importance. Buse et al. published the electrospray ionisation quadrupole time-of-flight hybrid tandem mass spectrometry of a homologous series of gemini surfactants [44]. The fragments and their corresponding *m*/*z* values for 12-6-12 are listed in Table 3.

## 4. Properties

### 4.1. Surface Activity in Aqueous Solutions

**Figure 6:**
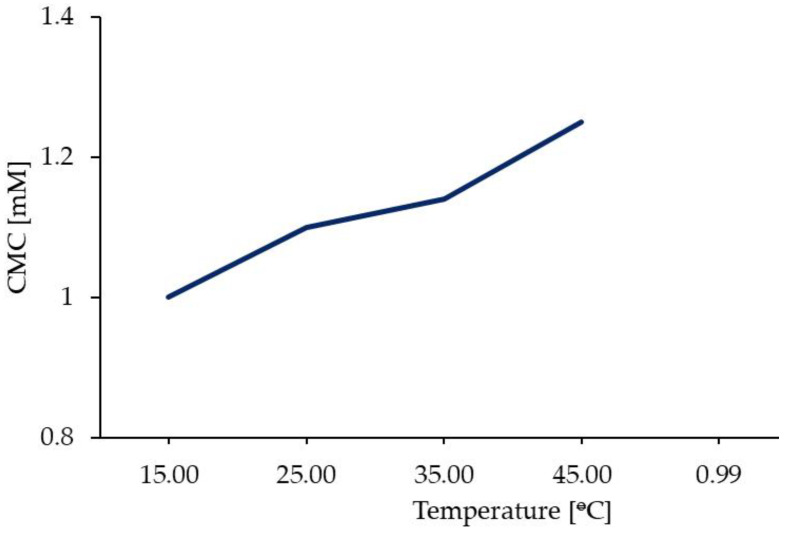
Plot of CMC versus temperature for 12-6-12.

Surfactants are molecules that lower the surface tension between two materials: a gas and liquid, a liquid and liquid, or a liquid and solid. Double quaternary ammonium salts, such as 12-6-12, are cationic surfactants. They possess long hydrocarbon chains (surfactant tails) and hydrophilic nitrogen groups (surfactant heads). The broad structural diversity of gemini surfactants renders it possible to obtain compounds with desirable HLB values and expected physicochemical properties. Gemini surfactants can interact very effectively with oppositely charged surfaces. Surfactant molecules in solution can form aggregates called micelles. The structure of micelles depends on many factors, including the solvent polarity. However, in every case, monomeric analogues require a higher number of molecules to form micelles than gemini surfactants [45,46,47]. 12-6-12 has much better surface properties than its single-chain analogue. The CMC, micelle ionisation degree (α), Gibbs free energy of micellisation (ΔG°_mic_), surface tension at CMC point (γ_CMC_), area per molecule (Å^2^), and number of molecules per nm^2^ for 12-6-12 and DTAB are listed in Table 4. The CMCs of 12-6-12 were two orders of magnitude lower than those of DTAB, regardless of the determination method. The ΔG°_mic_ value is more negative for 12-6-12 than for the monomer, suggesting a higher spontaneity of micellisation for the former. Generally, CMC values are highly dependent on temperature—the CMC values decrease with increasing temperature (Figure 6) [48]. A linear dependence with a high linear regression coefficient (r^2^ = 0.99) is observed.

The CMC values depend on the measurement method. The CMC values of 12-6-12 obtained by conductivity, fluorescence, and microcalorimetric measurements were 1.01, 1.09, and 0.89 mM, respectively [52]. Notably, the main unit responsible for the surface properties of 12-6-12 is the ammonium dication; the counterion is only of secondary importance (Table 5) [23]. Sulphates and nitrates have the lowest CMC values in this case, while bromide is the most active among the halides. Bromides can also be easily obtained via the S_N_2 reaction, without the need for ion-exchange columns [52].

12-6-12 forms micelles with a relatively small number of molecules. Wang et al. reported that 12-6-12 formed spherical micelles with 22 molecules [52]. This is in good agreement with the literature value [53]. Using small-angle neutron scattering (SANS) measurements, Burrows et al. confirmed that the radius of the spherical aggregates of 12-6-12 ranged from 1.74 ± 0.04 to 1.86 ± 0.04 nm [54].

Gemini surfactants undergo morphological transitions with increasing concentration, with the micelles changing from a spherical to an elongated shape. The concentration at which this morphological transition occurs is referred to as the second CMC and was reported to be 0.028 M for 12-6-12 by Graciani et al. [55]. At concentrations above the CMC, gemini surfactants tend to self-associate in water to form micelles whose characteristics depend on the nature of the surfactant as well as on the temperature. Additives affect the self-aggregation process and the features of the aggregates thus formed. This occurs owing to the variation in the chemical potential of the surfactant molecules in the bulk phase as well as in the micelles. The magnitude of an effect depends on the nature of the additive [55]. Currently, mixed micelles have become increasingly popular. These binary systems have been described for 12-6-12 with nonionic and zwitterionic surfactants [22,48,56].

### 4.2. Antimicrobial Properties

Gemini surfactants possess a broad spectrum of biocidal activity. The biocidal activity is initiated with the adsorption of quaternary ammonium cations on the negatively charged cell surface. Subsequently, long hydrocarbon chains diffuse through the bilayer of the cell, which increases the hydrophobicity of the bacterial cell membrane and triggers the disruption of the cytoplasmic membrane. Because of the damaged membrane, potassium ions and other low-molecular-weight cytoplasmic constituents are released, finally leading to the death of the microorganism cell. The biocidal activity of a microbiocide is usually determined by its MIC, that is, the minimal microbiocide concentration that inhibits the growth of microorganisms. MIC values are affected by several factors, such as the structure and concentration of microbiocide, time of contact, pH, temperature, and the presence of organic matter or other compounds [7,57,58,59,60]. The antimicrobial activity of gemini surfactants depends primarily on the length of the hydrocarbon substituent. Compounds with a dodecyl substituent are found to be the most effective microbicides [61,62]. The biocidal effectiveness of gemini surfactants depends on the type of microorganism. Gram-positive bacteria are more sensitive than Gram-negative bacteria. In general, the sensitivity of microorganisms to gemini surfactants decreases in the following order: Gram-positive bacteria > fungi > Gram-negative bacteria [7].

Sidenko et al. were the first to demonstrate the antimicrobial activity of 12-6-12 [21]. They found that 12-6-12 showed antibacterial effects at concentrations of 0.025% at 10 and 15 min and 0.01% at 20 and 25 min for *S. aureus* and *E. coli*, respectively [21]. Devinsky et al. studied the activity of gemini surfactants with different hydrocarbon chain lengths and found that the most effective microbicide was the one with a dodecyl substituent [63]. Ciganekowa et al. found 12-6-12 to be as effective as the commercially used disinfectants against different strains of Clostridium [64]. Many studies show that gemini surfactants have better antimicrobial activity than their monomeric analogues (Table 6) [32,65,66]. It is also worth noting that 12-6-12 is effective against planktonic forms at a very low concentration of 1.0145 mM, while it is effective at eradicating biofilms at a concentration of 0.29 mM [67]. Using scanning electron microscopy, Zhang et al. showed that this microbiome interacted with the bacterial cell membrane, disrupting the membrane integrity and ultimately killing the bacteria [32].

Gemini surfactants are also known to exhibit very high antifungal activity against yeasts and moulds in conidia and vegetative cells (Table 7) [7]. The MIC of 12-6-12 was 30 times lower than that of the single-chain analogue [65]. The mechanism of the antifungal action of 12-6-12 was studied by Koziróg et al. [68].

The antimicrobial activity of gemini surfactants against algae [70] and protozoa [39,71] has also been studied. Calas et al. showed that 12-6-12 inhibited the phospholipid metabolism of *Plasmodium falciparum* and exhibited good antimalarial activity. They reported the IC_50_ values of 12-6-12 and DTAB to be 0.22 and 0.5 µM, respectively [39].

Recent studies suggest that gemini surfactants exhibit antiviral activity. Khodsiani et al. reported that 12-6-12 and other gemini surfactants with long hydrocarbon chains showed the highest antiviral activity against influenza virus H1N1. This kind of compound may physically interact with hemagglutinin, a glycoprotein on the virus surface, at any dilution, indicating the ability of the compounds to inhibit viral attachment to the cell and the subsequent viral propagation. Apoptotic evaluation of the gemini surfactants highlighted their anti-apoptotic potential, especially for hydrophobic compounds [72].

### 4.3. Anticorrosion Properties

**Figure 7:**
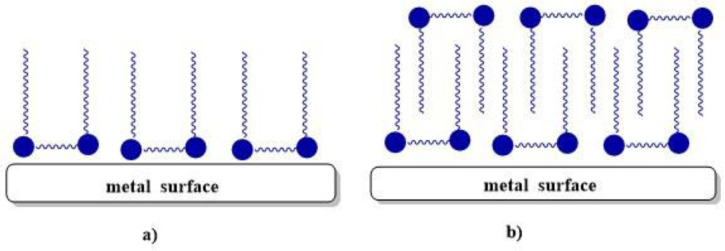
Adsorption model of 12-6-12 onto metal surface in acidic medium at concentrations (**a**) below CMC and (**b**) above CMC [73].

Recently, considerable attention has been paid to the use of gemini surfactants as corrosion inhibitors for metals and alloys. The mechanism of corrosion inhibition is based on the adsorption of the surfactant molecules onto the metal surface by displacing water molecules and the subsequent formation of a protective film. The mechanism can involve physical adsorption (electrostatic interaction), chemical adsorption (donor–acceptor interaction), or mixed adsorption [10]. The adsorption mechanism of 12-6-12 on a steel surface in an acidic medium depends on the surfactant concentration. The first phase for monolayer adsorption is formed below CMC, and the second phase for multilayer adsorption is formed at concentrations greater than CMC (Figure 7) [73].

12-6-12 shows excellent anticorrosion activity in acidic and salty media (Table 8). The anticorrosion activity is the highest and most effective at a concentration close to CMC [42,74].

The strong ability of 12-6-12 to adsorb onto a steel surface is responsible for its high anticorrosion activity. Analogous relationships have been demonstrated for metallic surfaces such as zinc surfaces [75].

12-6-12 exhibits antibacterial activity against *D. salexigens* and can act as a biocorrosion inhibitor even at low concentrations. After 12 days, the corrosion resistance was remarkably greater than that without an inhibitor, and no significant increase in sulfate-reducing bacteria was observed. The MIC against *D. salexigens* was 0.018 mM. An open-circuit potential experiment showed that this compound is an efficient biocide and corrosion inhibitor [74].

### 4.4. Interaction with Macromolecules

Gemini surfactants, as compounds containing ammonium cations, can interact with oppositely charged compounds and surfaces. Studies have been conducted on the adsorption of 12-6-12 on many non-organic surfaces, such as the silica–aqueous solution interface [53,76]; hydrophobised, hydrophilised, and untreated gold [24]; and a large group of aluminosilicate minerals [77,78]. The gemini surfactant can be used to tune the textual properties of zeolites or to endow biological properties to a material [78].

Mixtures of surfactants and polymers can be used to endow improved properties or novel functions that cannot be achieved using surfactants or polymers alone. Consequently, such mixtures have many practical applications, such as in paint and coating products, food processing, personal care formulations, enhanced oil recovery, and pharmaceutical formulations [79,80,81,82,83]. According to Han et al., the co-assembly of the poly(ethylene glycol)-b-poly-(glutamate sodium) copolymer with 12-6-12 leads to the formation of ordered nanosheets with a sandwich-like packing and an average size of 68 nm, exhibiting properties like those of superamphiphiles. The gemini molecules associate through hydrophobic interactions and constitute the middle part of the nanosheets, whereas the top and bottom of the nanosheets comprise hydrophilic polymer chains [79].

Gemini surfactants can interact effectively with biological macromolecules such as DNA [15]. This interaction must be strong enough to overcome the biological membrane barrier and weak enough to release DNA at the right location in the cell. The gemini surfactant is shown to bind and compact DNA efficiently and form a “lipoplex”. Lipoplexes can penetrate the outer membranes of many cell types to enter into the cytoplasm encapsulated within endosomes. The escape from the endosome may be controlled by changes in the aggregation behaviour of the lipoplex as the pH decreases. DNAs may be released from lipoplexes before their entry into the nucleus, where a new gene can be expressed with high efficiency [7,23]. Pisárčik and Devínsky studied the binding of gemini surfactants to DNA and found that the adsorption of DNA on 12-6-12 was the weakest, with only 7% of the surfactant molecules adsorbed [40,84]. Gemini surfactants can also interact strongly with proteins such as bovine serum albumin via electrostatic and hydrophobic forces [85,86].

## 5. Toxicity and Environmental Impact

Generally, amphiphiles are known to influence the organisation of lipid membranes, and surfactants have been extensively studied in systems involving interactions with lipid membranes [87]. Above a certain concentration, these compounds may exhibit undesirable properties, including toxicity. Therefore, it is very important to study the effects of substances on cells and living organisms before introducing them for use. Toxicity and CMC have been linked to gemini surfactants. Above a certain concentration, the toxicity of these compounds increases. It has also been shown that gemini surfactants are less toxic than their single-chain analogues [88].

Almeida et al. studied the cytotoxicity of gemini surfactants and suggested that the toxicity increases with increasing spacer length and that surfactants with longer tails are less toxic than those with shorter tails [87]. They chose the NCTC 2544 cell line, a human skin keratinocyte cell line, as a model of skin irritation. After 24 h of exposure to low concentrations of 12-6-12 (up to 10 mM), no significant cytotoxicity was observed in the cell line. However, at a 12-6-12 concentration of 50 mM, strong toxicity was observed [87]. The cytotoxicity and skin irritation profiles of 12-6-12 were also studied by Silva et al. [89]. These studies were performed in cultured human epidermal keratinocytes and human dermal fibroblasts. The skin integrity evaluation studies did not indicate relevant changes in the skin structure after the use of 12-6-12, while cytotoxicity studies established a relative cytotoxicity [89].

Koziróg and Brycki tested the haemolytic activity of 12-6-12 and DTAB in terms of the MIC against the morphotic elements of sheep blood [65]. 12-6-12 did not exhibit haemolytic activity at the highest MIC (0.029 μM/mL). A two-fold increase in the concentration lysed the erythrocytes slightly. Considerable disintegration of the erythrocyte membranes was observed at a 12-6-12 concentration of 0.58 μM/mL. DTAB, at the highest MIC of 1.01 μM/mL, caused slight haemolysis. In samples with 2.02 μM/mL of DTAB, a high degree (60%) of haemolysis was observed [65]. Thus, 12-6-12 shows lower haemotoxicity at the used concentration than its monomeric analogue. However, considering the reduced cytotoxicity of 12-6-12 compared to DTAB and that its required concentrations are many times lower than those of DTAB in order to obtain the same effect, its environmental impact will be much less than that of monomeric cationic surfactants.

Zhang et al. studied the cytotoxicity of gemini surfactants against a rat glioma cell line (C6) and human kidney cell line (HEK293) [32]. They presented the cytotoxic effect in terms of the IC_50_ values, which were 5.1 and 3.3 µM for C6 and HEK293, respectively [32]. Thus, 12-6-12 has potential applications in the medical field.

Research on the ecotoxicity of 12-6-12 and other gemini surfactants is sparse. Generally, these compounds are not readily biodegradable [90]. However, their degree of biodegradation can be increased using bacteria immobilised on alginate [91]. The degree of biodegradation of 12-6-12 determined by the CO_2_ headspace test was 0% [88]. Garcia et al. studied the aquatoxicity of 12-6-12 and DTAB against *D. magna*, and the IC_50_ values were 0.65 and 0.38 mg/L, respectively [88]. This confirms the previous conclusion that gemini surfactants are less toxic to aquatic organisms than their monomeric analogues.

## 6. Applications

Surfactants are ubiquitous, being key components in a diverse range of complex industrial processes and utilitarian products such as dispersants, solubilisers, emulsifiers, demulsifiers, foaming agents, wetting agents, disinfectants, corrosion inhibitors, antistatic agents, and viscosity modifiers. In the last few decades, significant efforts have been made for the synthesis of new gemini surfactants, fuelled by their remarkably improved physicochemical properties that can be achieved by the modification of structural factors [92]. Cationic gemini surfactants have wide applications due to their excellent surface activity [7,20]:

- in foaming, agrichemical spreading aids, and cleaning;
- from industrial to personal care applications;
- phase transfer catalyst;
- bleach activator;
- as hair conditioners and fabric softeners.

**Figure 8:**
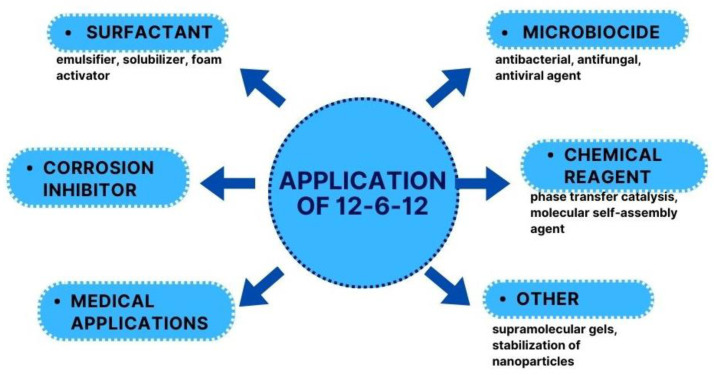
Applications of 12-6-12.

12-6-12 was first described 55 years ago, but the increased application interest of this compound can be defined in the 21st century. 12-6-12 can be used in several practical applications (Figure 8).

12-6-12 is characterised by excellent surface, antimicrobial, and anticorrosion properties. As a compound that can be obtained economically and ecologically, it is an ideal product that has many industrial applications, including bioapplications (Table 9).

Moreover, 12-6-12 can be used in chemical synthesis for the preparation of supramolecular gels [101] and as a nanoparticle stabiliser [28,41,102], an interfacial transfer catalyst [30,31,103,104,105], and a molecular self-assembly agent [106].

In conclusion, quaternary ammonium gemini surfactants are known for their multifunctional utility properties such as antimicrobial, surfactant, and anticorrosion properties. 12-6-12 has all the above mentioned properties of gemini surfactants and can be obtained in a one-step reaction, which is an indisputable and economically justified advantage over other gemini surfactants. Compared to its monochain counterpart, it shows better surface, antimicrobial, and anticorrosion activity and is characterised by a lower toxicity. The use of this compound in concentrations much lower than DTAB while providing the same utility effect makes 12-6-12 a candidate in many applications. Initially, this compound was used in applications as typical quaternary ammonium salts as an interfacial transfer catalyst. Currently, this compound finds potential applications in many different areas of life. Particularly noteworthy is the testing of this compound in biomedical applications. Due to its anticorrosive, biocidal, dispersing, and detergent properties, 12-6-12 can be used as an additive to car fuels and as an anticorrosion and antimicrobial agent in oil pipelines, smart anticorrosion coatings, etc. Hence, the possibility of producing it in large quantities in a one-step, waste-free synthesis is a huge advantage over other gemini surfactants.

## Figures and Tables

**Table 1:** ^1^H NMR chemical shifts (δ, ppm) of 12-6-12 in different solvents.

Proton	δ		
	D_2_O ^1^	CDCl_3_ ^2^	CD_3_OD ^3^
a (6H)	0.86	0.85	0.86
b (36H)	1.37–1.28	1.33–1.19	1.37–1.28
c (4H)	1.77	1.89	1.77
d (4H)	3.35	3.68	3.34
e (12H)	3.12	3.35	3.12
f (4H)	3.32	3.42	3.31
g (4H)	1.71	1.69	1.71
h (4H)	1.45	1.35	1.45

^1^ [35]; ^2^ [38]; ^3^ [42].

**Table 2:** ^13^C NMR chemical shifts (δ, ppm) of 12-6-12 in different solvents.

Carbon	δ	
	D_2_O ^1^	CDCl_3_ ^2^
a	15.83	13.84
b	33.96–31.48	31.60–29.17
c	24.41	22.38
d	64.84	64.38
e	53.51	50.73
f	64.72	63.81
g	23.86	21.51
h	27.10	24.42

^1^ [35]; ^2^ [38].

**Table 3:** Molecular and fragmentation ions and their corresponding *m*/*z* value for 12-6-12.

Ions	*m*/*z*
[M]^2+^	255.30
[M-C_12_H_25_]^+^	341.41
[M-C_12_H_24_]^2+^	171.20
[M-C_12_H_24_-C_12_H_24_-C_2_H_5_N]^+^	128.12

**Table 4:** Various micellisation and thermodynamic parameters of DTAB and 12-6-12.

Parameters	DTAB	12-6-12
CMC (conductometric) [mM]	15.4 ^1^	0.98 ^2^
CMC (tensiometric) [mM]	15.0 ^3^	0.85 ^3^
A	0.29 ^3^	0.46 ^3^
ΔG°_mic_ [kJ/mol]	−35.0 ^4^	−59.8 ^4^
γ_CMC_ [mN/m]	37.5 ^3^	37.7 ^3^
Å^2^ [nm^2^]	62 ^3^	108 ^3^
Number of molecules per nm^2^	1.61 ^3^	0.93 ^3^

^1^ [49]; ^2^ [50]; ^3^ [24]; ^4^ [51].

**Table 5:** CMCs of different anions of 12-6-12.

Anion	CMC [mM]
SO_4_^2−^	0.68
NO_3_^−^	0.89
Br^−^	0.98
Ac^−^	1.10
Cl^−^	1.33
F^−^	1.84

**Table 6:** Antibacterial activity of DTAB and 12-6-12.

Bacteria	MIC [mM]	
	DTAB	12-6-12
Gram-positive:		
*S. aureus*	-	0.008 ^1^
	0.044 ^2^	0.0028 ^2^
	0.252 ^3^	0.0036 ^3^
*C. perfringens*	-	0.04 ^4^
Gram-negative:		
*E. coli*	-	0.052 ^1^
	0.36 ^2^	0.0868 ^2^
*P. aeruginosa*	0.126 ^3^	0.0073 ^3^
*A. lannensis*	0.127 ^5^	0.0073 ^5^

^1^ [63]; ^2^ [32]; ^3^ [65]; ^4^ [64]; ^5^ [66].

**Table 7:** Antifungal activity of 12-6-12.

Fungus	MIC [mM]
*A. niger*	0.12 ^1^
*P. chrysogenum*	0.06 ^1^
*C. albicans*	0.015 ^1^ 0.022 ^2^
*A. brasiliensis*	0.12 ^3^

^1^ [35]; ^2^ [63]; ^3^ [69].

**Table 8:** Parameters in the absence and presence of different concentrations of 12-6-12 after immersion for 24 h (corrosion rate = CR; inhibition efficiency = IE).

Concentration of Surfactant [mM]	Material	Corrosive Medium	CR [mm/year]	IE [%]	Ref
0	Stainless steel	3 M HCl	19.74	-	[42]
0.1			3.71	84	
0.5			0.92	95	
1			0.71	97	
5			0.65	97	
0	Mild steel	3.5% NaCl	1.39	-	[74]
0.01			0.068	95.2	
0.1			0.021	98.5	
1			0.019	98.7	
2			0.010	99.3	

**Table 9:** Potential bioapplications of 12-6-12.

Application	References
Enhanced drug delivery	[87,89]
Drug carrier	[93]
Stabilization of drugs	[94]
Detection of DNA	[33]
Nonviral gene delivery agent	[44,95,96,97]
Eradication of biofilm	[66,67]
Filtering nonwovens	[78]
Bioactive cellulose products	[98,99]
Addition to DNA filtration	[100]

## Data Availability

No new data were created or analysed in this study. Data sharing is not applicable to this article.

## Sample Availability

A sample of the compound is not available from the authors.
